# Current State of COVID-19 Pandemic in Africa: Lessons for Today and the Future

**DOI:** 10.3390/ijerph18199968

**Published:** 2021-09-22

**Authors:** Godwin Attah Obande, Ahmad Ibrahim Bagudo, Suharni Mohamad, Zakuan Zainy Deris, Azian Harun, Chan Yean Yean, Ismail Aziah, Kirnpal Kaur Banga Singh

**Affiliations:** 1 Department of Medical Microbiology and Parasitology, School of Medical Sciences, Health Campus, Universiti Sains Malaysia, Kubang Kerian 16150, Malaysia; obandega@student.usm.my (G.A.O.); aamdee96@gmail.com (A.I.B.); zakuan@usm.my (Z.Z.D.); azian@usm.my (A.H.); yychan@usm.my (C.Y.Y.); 2 Department of Microbiology, Faculty of Science, Federal University of Lafia, PMB 146, Lafia 950101, Nigeria; 3 Department of Microbiology, Faculty of Life Sciences, Kebbi State University of Science and Technology, PMB 1144, Aliero 863104, Nigeria; 4 School of Dental Sciences, Health Campus, Universiti Sains Malaysia, Kubang Kerian 16150, Malaysia; suharni@usm.my; 5 Medical Microbiology Laboratory, Hospital USM, Kubang Kerian 16150, Malaysia; 6 Institute for Research in Molecular Medicine (INFORMM), Health Campus, Universiti Sains Malaysia, Kubang Kerian 16150, Malaysia; aziahismail@usm.my

**Keywords:** COVID-19, Africa, SARS-CoV-2, coronavirus, infection, healthcare system

## Abstract

This study is a cross-sectional, observational analysis of the COVID-19 pandemic in Africa, to understand the progression of the disease across the continent. Published data on COVID-19 from 20 January 2020 to 21 June 2021 were obtained and analyzed. Case fatality ratios, as well as case growth rates and other indices were computed. On 21 June 2021, a total of 178,210,532 confirmed cases and 3,865,978 deaths had been recorded worldwide. While the Americas recorded the highest number of cases, Southern Africa recorded the majority of African cases. Fatality rate since from 20 February 2020 to 21 June 2021 was highest in the Americas (2.63%) and low in the South Eastern Asia region (1.39%), globally increasing from 2.17% at the end of January to 6.36% in May 2020 and decreasing to a range between 2.14% to 2.30% since January 2021. In Africa, the infection rate per 100,000 persons was up to 3090.18, while deaths per 100,000 and case fatality ratio were as high as 119.64 and 5.72%, respectively, among the 20 most-affected countries. The testing rate per million population was highest in Botswana (512,547.08). Fatality appears to be increasing in some regions of Africa. The rate of infection and fatality in Africa could still likely take an upward turn. Strict control measures are required, considering the continent’s weak healthcare systems.

## 1. Introduction

On 31 December 2019, Chinese authorities reported cases of pneumonia in Wuhan, Hubei Province in China, whose causal agent was unknown until 7 January 2020, when it was identified and confirmed to be a novel coronavirus. Within four days (31 December 2019 to 3 January 2020), 44 cases had been identified, and between 13 to 20 January 2020, Thailand, Japan, and the Republic of Korea each reported first cases of the virus. Following the spread of the virus to different parts of the world, the outbreak was declared a public health emergency of international concern (PHEIC) by the World Health Organization (WHO) on 30 January 2020 [1]. The SARS-CoV-2 outbreak is the sixth to be so declared by the WHO, the others being H1N1 influenza (2009), Ebola (2014 and 2019 in West Africa and the Democratic Republic of Congo, respectively), polio (2014), and Zika (2016) [2].

The virus was first widely known by the name ”Wuhan coronavirus” or the ”Chinese coronavirus”. However, the name of the disease was changed by the WHO, from 2019 novel coronavirus (2019-nCoV) to COVID-19 (coronavirus disease 2019) on 11 February 2020, while the virus is known as severe acute respiratory syndrome coronavirus-2 (SARS-CoV-2). The name follows the recognized relatedness of the new viral agent with the severe acute respiratory syndrome coronaviruses (SARS-CoVs), which belongs to the *severe acute respiratory-syndrome-related coronavirus* species found in humans and bats [3].

Initial reports of SARS-CoV-2 had linked the outbreak to a market for seafood and wild animal meat, suggesting animal to human transmission [4]. However, rapid person-to-person spread of SARS-CoV-2 by both symptomatic and asymptomatic patients through droplets and aerosols from coughing and sneezing, as well as direct contact, has been reported [5]. Symptoms of the viral disease can range from fever, dry cough, myalgia, fatigue, dyspnea, and anorexia at the start, to atypical symptoms, such as diarrhea and nausea, and complications such as acute respiratory distress syndrome, arrhythmia, and shock, which may appear between 2 and 14 days after infection.

The continent of Africa appeared safe from the SARS-CoV-2 pandemic at the onset of the disease in China and a few other countries. By the 14 February 2020, the Northern African country of Egypt reported Africa’s first confirmed case of COVID-19. The first index case in Africa was a foreigner who was visiting Egypt [6]. Following this, Algeria, also in Northern Africa, and Nigeria in Western Africa, both reported their first cases on 25 and 27 February 2020, respectively [7]. Both cases were Italian nationals who had travelled to the affected countries at the time. In March 2020, new cases were also reported in the Western African countries of Senegal (1st), Togo (6th), Burkina Faso (9th), and Ghana (12th); the Northern African countries of Tunisia and Morocco (2nd); the Southern African nation of South Africa (5th); and Central African countries of Cameroon (6th), Democratic Republic of Congo (10th), and Ivory Coast (11th). On 13 March, the first cases were also reported in Eastern Africa (Ethiopia, Kenya, and Rwanda), Southern Africa (Namibia), Central Africa (Gabon), Northern Africa (Mauritania), and Western Africa (Guinea) [8]. Sahrawi republic, the last African country to report a positive COVID-19 case, saw its first reported infection on 24 July 2020. The number of cases on the continent has since increased, with its attendant effect on livelihood and national economies. The third sustainable development goal (SDG) focuses on good health and well-being. To achieve this, a pandemic such as COVID-19 requires continuous study of trends and other transmission indicators that will avail abundant information for effective policy formulations, especially in a continent faced with limited resources in almost every sector. An evaluation of control measures instituted on the continent will also be beneficial to controlling this and future pandemics on the continent. This study aimed to examine the current situation of the COVID-19 pandemic from the first reported cases in Africa and other regions of the world, to enable a better understanding of its trends across the various regions of the African continent. The response of African nations to the outbreak are also analyzed.

## 2. Materials and Methods

### 2.1. Sources of Data and Study Design

The study is a cross-sectional, observational, retrospective analysis of publicly available COVID-19 data from verified sources, covering 20 February 2020 to 21 June 2021. Published research and review articles on COVID-19, which reported findings from different parts of the world at the start of the disease, were obtained from the Web of Science Core Collection (Clarivate Analytics), ScienceDirect (Elsevier), Google Scholar, and PubMed (National Library of Medicine) databases.

Data for this study were obtained from the COVID-19 daily situation reports of the WHO (available at https://www.who.int/emergencies/diseases/novel-coronavirus-2019/situation-reports (accessed on 22 June 2021 and https://covid19.who.int/ (accessed on 18 August 2021) and the Africa CDC Dashboard for COVID-19 (available at https://africacdc.org/covid-19/, accessed on 22 June 2021), beginning from 20 January 2020. The African CDC obtains these data from member countries and the Collaborating Center of the region. By the AU resolution CM/Res.464QCXVI of 1976, Africa is divided into five distinct geographical regions: Central Africa, Eastern Africa, Northern Africa, Southern Africa, and Western Africa. This classification was adopted during the computation of COVID-19 data for the various regions of Africa.

Population estimates were obtained from the United Nations population records [9]. Population data for some countries not reported by the United Nations population records were obtained from The World Bank [10] and the official websites of the respective countries. [11,12,13] Global daily confirmed cases and daily deaths were collated. In addition, number of recoveries, number of tests performed within the overall population, and population of the twenty most affected countries in Africa were also computed.

Data on the number of tests performed by countries daily or weekly were obtained from the official websites or social media accounts of the country’s agency responsible for providing updates on the pandemic. Some testing data were also collated from the open-source Statistics and Research records of Ritchie et al. [14] hosted on ourworldindata.org. Where the cumulative number of tests performed from the start of the pandemic to June 2021 was not readily available, daily reported test data were pooled together until June 2021. Data reported beyond 21 June 2021 were not included in the computation and analysis for the number of tests performed.

### 2.2. Data Computation, Descriptive Analysis, and Visualization

The collated data were organized into Microsoft Excel 2016 (Microsoft Corporation), which was also used to compute descriptive statistics such as percentages, total cases, deaths, tests per million, and case fatality ratio (CFR) for the twenty most affected African countries as of 21 June 2021. Stack columns (100%), clustered columns, and line graphs were plotted using Microsoft Excel 365 (Microsoft Corporation, Redmond, WA, USA). The desktop version of Quantum gis software (Version 3.18.3-Zurich, Free Software Foundation, Boston, MA, USA) was used for the mapping of data and map generation. Global daily case growth rates were computed as a percentage of the ratio between daily reported cases and cumulative daily cases. The CFR, which represents the mortality per absolute number of cases, was computed as a percentage of total deaths divided by the total number of confirmed cases. The cases and deaths per 100,000 people account for mortality resulting from COVID-19 infections within the general population of both healthy and infected individuals and was computed as a percentage of the total cases or deaths over the entire country population or region as obtained from the United Nations population size records [9] and other sources [11,12,13]. Similarly, testing rates were computed as the total number of tests performed in 1,000,000 (or 100,000) persons relative to each country or region’s total estimated population size.

## 3. Results

### 3.1. COVID-19 Cases, Deaths, and Testing Rates in Different World Regions

**Figure 1:**
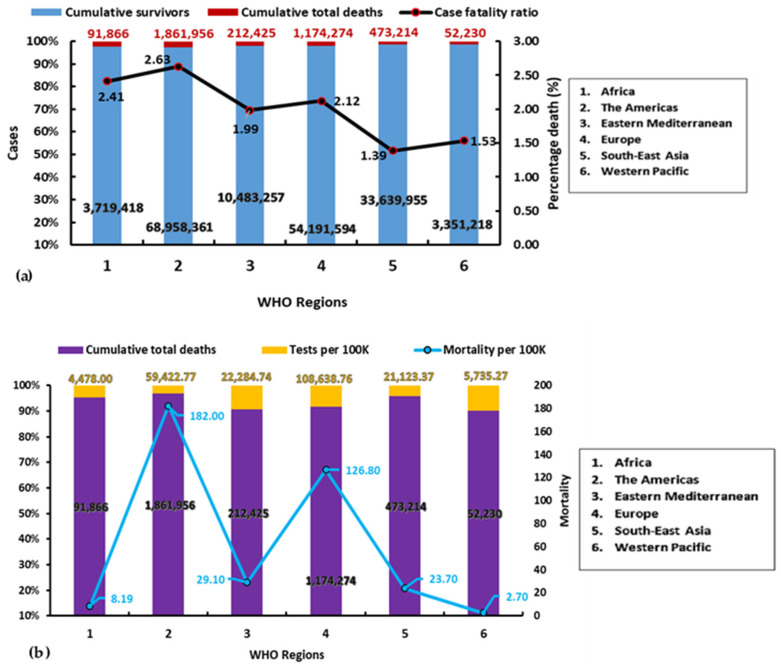
(**a**) World-wide COVID-19 cases and deaths according to regions, based on WHO data of 21 June 2021. (**b**) COVID-19 deaths, mortality per 100,000 persons and test rate per 100,000 persons according to regions, based on data up to 21 June 2021.

As of 6 p.m. CEST on 21 June 2021, the total number of confirmed COVID-19 cases in the world stood at 178,210,532 (344,340 new cases) and a total of 3,865,978 deaths (8002 new deaths). According to the WHO report, the Americas region had recorded the highest number of confirmed cases (70,820,317) and deaths worldwide (Figure 1a,b). On the contrary, the Western Pacific region had the fewest confirmed cases (3,418,284) with 52,505 deaths. The Americas region had the highest fatality rate of 2.63% while Southeast Asia region had the lowest fatality rate (1.39%). The CFR of the African region (2.41%) was the second-highest within the period, a value higher than the regions of Europe (2.12%), Western Pacific (1.53%), and Southeast Asia (1.39%).

Based on population estimates from the United Nations population records [9], the World Bank [10] and official country sources [11,12,13], mortality rates per 100,000 persons in the various WHO regions (Figure 1b) showed that the Americas region with an estimated population of 1,022,775,204, which is the fourth-largest among the WHO regions, experienced the highest mortality rate per 100,000 persons (182.0), ahead of the region of Europe (126.8) with an estimated population of 934,064,442, which is the fifth-largest population estimate among the WHO regions. On the other hand, the Eastern Mediterranean region with the smallest population estimate (730,822,202) experienced a higher rate of mortality per 100,000 persons (29.1) than the Southeast Asian (23.7) and African (8.19) regions with an estimated population of 2,021,386,630 and 1,121,335,427, respectively. Despite having a larger population estimate, only less than that of the Southeast Asian region, the Western Pacific region with an estimated population of 1,932,593,585 recorded the lowest mortality rate per 100,000 persons among the WHO regions of the world, being 2.7.

COVID-19 testing rates based on population estimates and available data on tested samples showed that the African region with the 3rd largest population had conducted the lowest number of tests per 100,000 persons (4478.00) since the pandemic began. The Western Pacific region with the second-largest population had conducted 5735.27 tests per 100,000 persons, behind the regions of the Americas (59,422.77), Southeast Asia (21,123.37) and Eastern Mediterranean (22,284.74). The region of Europe had conducted the largest number of tests per 100,000 persons since the onset of the pandemic, being 108,638.76.

### 3.2. Global Daily Cases, Deaths, Growth Rate, Case Fatality Ratio, Mortality, and Test Rates

**Figure 2:**
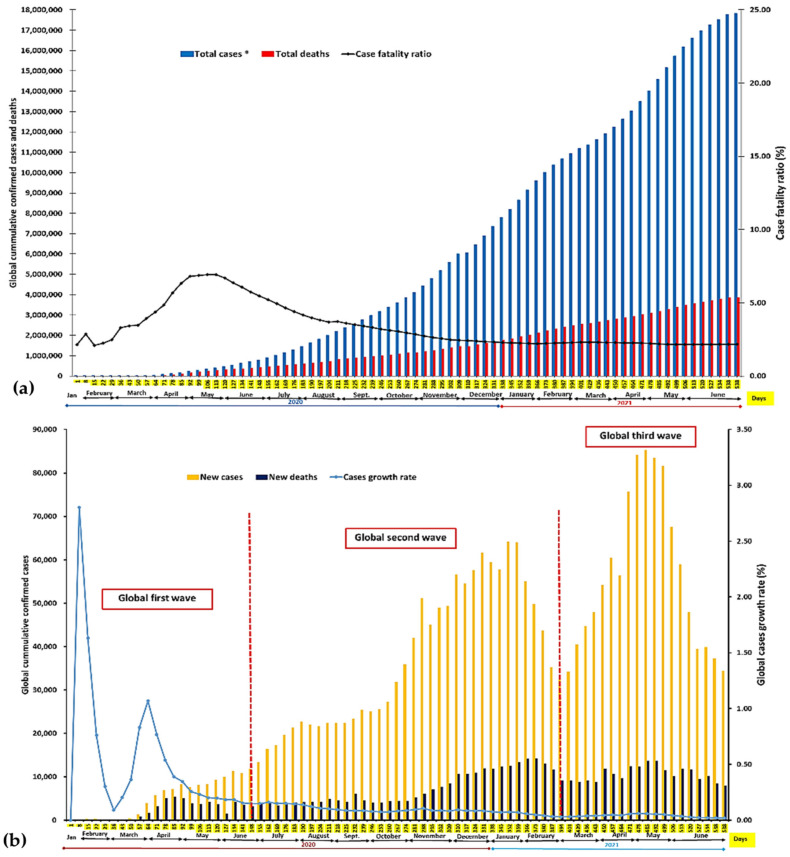
(**a**) Temporal global COVID-19 cumulative cases, cumulative deaths, and CFR based on WHO data of 21 June 2021. * Actual values have been adjusted downwards by 10 units to fit both data into the y-axis. (**b**) Temporal global COVID-19 daily new cases, daily new deaths, and daily case growth rate based on WHO data of 21 June 2021.

The temporary daily cumulative confirmed cases and deaths are as shown in Figure 2a. Daily cumulative total cases increase as new cases are reported all over the world. Similarly, daily cumulative deaths increase with rising reports of mortality in different parts of the world. The global case fatality ratio (CFR) rose from a low 1.91% in January 2020 to 6.36% on 26 May 2020. The CFR rose steadily from February 2020 and continued the trend throughout March and April 2020. A considerable rise in CFR was observed from 5.08% to 7.12% over about one month from April through May 2020. Globally, more daily deaths were recorded within this period. The global CFR began a downward trend from June 2020 and has declined reasonably since then. As of 21 June 2021, the global CFR was 2.17%. The peak CFR observed over the period has remained unsurpassed as of June 2021. Although new cases are reported daily, the growth rate of daily new cases globally showed a slightly different path (Figure 2b). The first wave of cases started when the disease spread from China to other parts of the world.

The period coincided with the Chinese New Year celebration, during which Chinese citizens travel from China to different destinations around the world. A spike in cases was observed in July 2020, which rose to a peak in January 2021, representing the second wave globally. A third wave of infections erupted in late February 2021 and lasted through May to June 2021. The highest number of cases reported in a single day globally was on 29th April 2021, when over 900,000 confirmed cases were recorded worldwide (Figure 2b). This figure surpassed an earlier all-time high recorded on 22nd April. The highest number of deaths recorded in a day globally since the pandemic began was observed during the second wave in January 2021. Over 14,000 deaths were reported between the 20 January and 24 January, with the highest recorded on 27th January 2021.

Daily deaths within the third wave were higher than those observed within the first wave. The growth rate of new cases rose sharply in late February but fell steadily through the beginning of March. The growth rate peaked between 20 and 23 March 2020 and began to fall again through the end of March 2020, April, and May 2020. The daily case growth rate also showed a slight increase at the start of each wave experienced during the period. However, the daily growth rate in new cases remained lower in June 2021 than at any time since the pandemic began.

### 3.3. Comparison of COVID-19 in Countries and Regions of Africa

**Figure 3:**
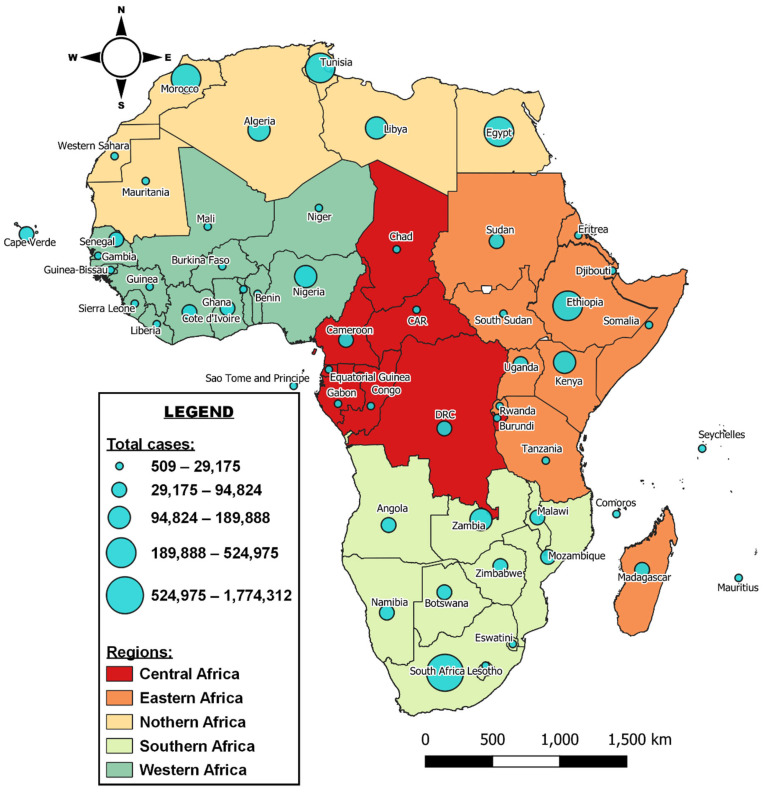
Overview of COVID-19 in Africa based on AU regions and Africa CDC data of 21 June 2021. Data source: https://africacdc.org/covid-19/ (accessed on 22 June 2021).

Based on the 55-member country listing by the African Union (AU), the total number of confirmed cases recorded on the African continent as of 26 May 2020 differed from that reported by the WHO and was 3,811,284 cases with 91,866 deaths. This is because in the WHO regional classification, Egypt, Djibouti, Tunisia, Libya, Sudan, and Morocco are grouped under the Eastern Mediterranean Region but are part of Northern and Eastern African regions in the AU grouping (Figure 3). The nation of South Africa has had the highest number of cases, having reported over a million confirmed cases as of 21 June 2021. Morocco had the second highest number of cases (526,737), closely followed by Tunisia, Egypt and Ethiopia with 385,428, 277,797 and 275,318 cases, respectively. Libya, Kenya, Nigeria, Algeria, and Zambia had recorded over 100,000 cases while Western Sahara and Tanzania reported fewer than 1000 cases as of 21 June 2021.

**Figure 4:**
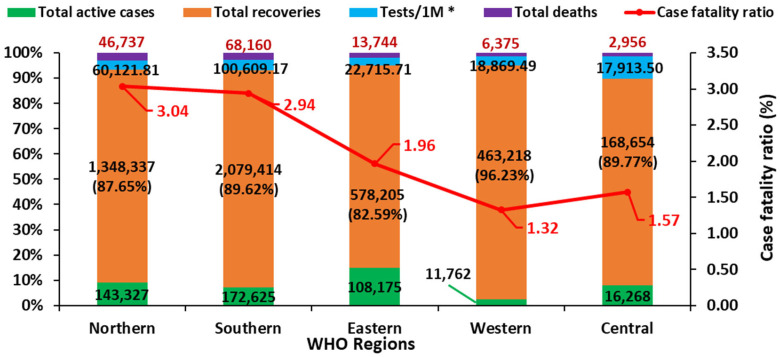
Overview of COVID-19 cases, death recoveries, test rates, and CFR in regions of Africa based on AU geographical grouping and Africa CDC data of 21 June 2021. * Tests per 1 million persons in the total population.

The Southern African region accounted for the highest number of cases with 2,320,199 confirmed cases and 68,160 deaths (Figure 4). The number of infections was lowest in the Central African region (187,878 cases). The CFR was lowest in Western Africa (1.32%) and highest in Southern Africa (3.04%). Western Africa record the highest recovery rate (96.23%) between 2020 and 21 June 2021, ahead of Central Africa (89.77%), Southern Africa (89.62%), Northern Africa (87.65%), and Eastern Africa (82.59%) (Figure 4). In terms of testing rates, the Southern African region had conducted the largest number of tests per 1,000,000 persons (tests/1M), amounting to 100,609.17. Northern, Eastern, and Western Africa conducted 60,121.81, 22,715.71, and 18,869.49 tests/1M, respectively, ahead of Central African region with the lowest test/1M (17,913.50).

Among the twenty most affected countries in Africa (Table 1), South Africa had the highest number of confirmed cases as on 21 June 2021, with 1,832,479 confirmed cases. The AU CDC data also showed that Egypt had the highest CFR (5.72%) as of 21 June 2021. Despite having the highest number of cases, South Africa’s CFR (3.21%) was much smaller than that of Libya (5.66%), Zimbabwe (3.99%) and Tunisia (3.66%).

As of 21 June 2021, Tanzania in Eastern Africa recorded the lowest number of cases (509). On the other hand, Cape Verde had the highest number of cases per 100,000 (5297.17), while Tunisia had the highest deaths per 100,000 (1537) in Africa and ranked fourth highest in terms of confirmed cases on the continent among countries with the most cases in Africa. In terms of testing capacity among the 20 most affected countries, Botswana conducted 525,549.17 tests per million population, the highest on the African continent by any country since the pandemic began (Table 1). Botswana, with the lowest population among the 20 most affected countries, had performed a larger number of tests per million persons than Nigeria (10,997.53) which has the largest population on the African continent. As of 21 June 2021, testing capacities among the top 20 countries with the highest number of confirmed COVID-19 cases in Africa ranged from 5251.78 per million in Algeria to 207,956.78 per million population in South Africa, which has conducted more than 12 million tests.

## 4. Discussion

COVID-19 transmission rates differ from country to country. This variation in numbers could be influenced by population characteristics such as socioeconomic status, nutrition, age, race, population density, genetic composition, lifestyle, or presence or absence of comorbidities [15]. These characteristics vary from one continent to another. The United States of America (USA), as of 21 June 2021, had the highest number of confirmed cases (33,190,195). The high growth rate in new cases observed within the first ten days of the disease in almost all the regions may suggest the absence of adequate, effective prevention and control measures. As understanding of the virus and its epidemiology increased, the application of control and preventive measures began to yield results in many countries, resulting in a decrease in daily case growth rates, which would have been worse without such efforts. Being a measure of how the number of cases changes from the previous value to the newly reported, the cases growth rate is influenced by the magnitude of increase or decrease in new cases at a given time. Restrictions in both local and international travel and prohibition of mass gatherings and other non-pharmaceutical measures did slow transmission from one person to another and hence influenced the daily case growth rate. Three different waves of infection have been experienced worldwide since the start of the pandemic, with each wave displaying its unique characteristics. Studies suggest that the evolution of SARS-CoV-2 genetic variants has influenced the transmission characteristics of each wave. For instance, strains of SARS-CoV-2 circulating during all three waves in Hong Kong, China were found to be different from each other [16]. These variants are broadly classified as variants of concern or variants of interest; each group differs in their transmissibility characteristics. The third wave, which greatly devastated India between April and May 2021, coincided with the emergence of the now widely spreading Delta variant of concern (B.1.617.2), which is reported to be 60% more transmissible than the Alpha variant (B.1.1.7) of concern first found in the UK during the second wave (December 2021). The Delta variant also more than doubles the likelihood of hospitalization compared to the Alpha variant, which was more predominant during the second wave [17]. Evidence from the daily new cases showed that the highest infection rates since the pandemic began were recorded during the third wave, largely due to the Delta variant. The second wave of infections was more aggressive in Africa than the first wave, which progressed much more slowly. The first wave peaked in mid-July on the continent, but some African countries were still experiencing their first wave as of December 2020 [18]. The second wave began in most African countries in December 2020, with a reported increase in daily cases up to 30% higher than the previous wave. A surge in cases and deaths was observed in South Africa’s second wave caused by the Beta variant of concern (B.1.351), first discovered in that country. This variant can cause reinfection in people who recovered from previous variants and was less affected by some vaccines [19]. Currently, there are indications of the spread of the Delta variants into parts of Africa due to the surge in cases reported in countries such as Malawi, Senegal, Namibia, Nigeria, Rwanda, South Africa, DRC, Uganda, and Tunisia since the beginning of July 2021. With fewer than 20 million fully vaccinated individuals on the continent as of early July 2021, the seemingly low cases in Africa may skyrocket with the spread of this variant which is reported to affect younger adults more than older variants [17,20].

When considered on general terms and the number of days since first infection, the number of confirmed cases and recorded deaths due to COVID-19, and the records of daily case growth rate on the African continent has been significantly lower than all the other regions of the world. As of 21 June 2021, the number of cases in Africa only slightly exceeded that of the Western Pacific region. Factors such as the larger proportion of young people in the population, a warm climate, the use of BCG vaccinations in tuberculosis patients, and cross-protective immunity from related coronaviruses and parasitic infections which are prevalent in Africa, are thought to have played a role in the low number of cases recorded so far on the continent [21,22]. Mortality has been higher in populations with more older people than younger people, as was observed in countries such as Italy. As of 2019, persons aged 65 years or older constituted 23% of the country’s population in Italy. In Italy and China, the mortality rate increased as age increased, with more deaths recorded among people 60 years or above [23].

Other factors could also be responsible for the low cumulative cases and growth rate in new cases within Africa. Firstly, many countries in Africa are not conducting enough tests compared to countries in other regions of the world. For instance, the number of tests conducted since the pandemic in many African countries is far fewer than that performed within the same period in the USA [24] and some other developed countries. With over 1 billion people, the continent had tested fewer than 3 million people as of November 2020 [25]. As also supported by our data, the testing rate per 100,000 persons is lower on the African continent. The implication of these low testing rates in many African countries is the risk of unidentified and undocumented community spread that could result in a prolonged presence of the disease on the continent. With increased testing, milder cases can be detected earlier, thereby lowering mortality resulting from the disease. Deaths that are due to COVID-19 infections could easily be recorded as being natural or of unknown cause where no test was performed on a patient prior to death. This could jeopardize the concerted efforts being expended towards the total control of the pandemic worldwide. In addition, the number of tests performed influences the number of cases that can be identified over a period of time, judging from the experiences of other nations [26]. Data on testing rates are scarce or not updated regularly in some countries in Africa and other regions. This may explain why the number of cases reported in some African countries remains low at this time and clearly influences what is known about the true incidence of COVID-19 in Africa [27,28]. To have a true picture of the disease incidence in Africa, increased testing rates across the continent is of paramount importance. Many African countries have experienced a shortage of testing kits, making large-scale testing a problematic task. Despite the limited economic and health resources on the continent, the Partnership to Accelerate COVID-19 Testing reportedly secured 90 million test kits for use on the continent as of mid-2020 [29]. There is clearly a need for nations in Africa to increasingly sustain efforts to develop research and manufacturing capacity to mitigate the challenges the continent faces in medical diagnostics and consumables. Innovative ways of tackling challenges in health systems must become a top priority on the continent, and some African countries have begun evolving ways of achieving this. For instance, Ghana pioneered an innovative way of driving faster testing earlier on in 2020, by using drones to fly samples from remote areas to testing centers in the capital [30]. This made Ghana the first country to apply this technology in the fight against COVID-19. Such efforts must be sustained and vigorously improved upon to drive the continent’s required revolution for improved well-being.

Secondly, many infected people with symptoms of the disease may not be aware or may refuse to present themselves for testing and treatment, probably due to the fear of stigmatization. For instance, widespread discrimination against Asians had followed the origination of disease from China [31]. Before COVID-19, stigmatization of people with conditions such as HIV had been prevalent in many parts of Africa [32]. Additionally, many cases in Africa could be asymptomatic and may hence, not be reported or detected. A previous report showed that four-fifths of cases in China were asymptomatic [33]. Asymptomatic patients can transmit the disease to healthy individuals even when no symptoms are displayed [34].

Reduction in the growth rate of daily new cases, though irregular in pattern, could be due to the stringent measures implemented by many countries. Public places such as airports, schools, markets, seaports, religious centers, and social and recreational centers were shut down. Ethiopian Airlines, the largest in Africa, also swiftly suspended flights in response to the pandemic. Social distancing and the use of alcohol-based hand sanitizers was highly promoted as well. The initial reaction by some countries was the screening of passengers at points of entry even before the first case was reported on the continent. As cases increased across Africa, some countries closed their borders, imposed movement restrictions, and set up isolation centers where confirmed cases were held, as well as testing laboratories for suspected cases. Impressively, a meeting of emergency responders across countries in Africa was held in Senegal earlier in February 2020 to build participating nations’ capacity in COVID-19 diagnosis [35]. From the first two laboratories for COVID-19 testing as of early February (Senegal and South Africa only), the number of testing laboratories rose to 43 by 7 March 2020 [36]. As of 15 April 2020, 47 countries in the WHO African region [37] and the 55 AU member countries all had COVID-19 testing capabilities [18]. Three days after Nigeria announced the index case of COVID-19, the genome sequence of the SARS-CoV-2 obtained from the sputum of an infected Italian visitor was made public. This was the first effort by any African nation at providing genomic information on the SARS-CoV-2 by in-country analysis [38,39]. Proactive preparations were also seen in Zambia, South Africa, and Uganda with the setting up of treatment centers, laboratories, and screening equipment such as thermal scanners at entry ports and training for health and aviation personnel by the Africa CDC with support from United States CDC, the WHO, and the International Civil Aviation Authority [35]. Awareness campaigns that increased people’s knowledge about the virus and its transmission and infection prevention strategies could also have played a critical role in reducing the growth rate of daily new cases. The trend in cases and deaths in Africa as of 21 June 2021 appears to confirm the effectiveness of these measures in curbing the pandemic.

Among the 20 most-affected countries in Africa, there appears to be a trend in the number of reported cases relative to the geographical location on the continent and level of exposure to the international community. As of 21 June 2021, the highest numbers of cases (in descending order) had been reported in South Africa, Morocco, Tunisia, Egypt, Ethiopia, Libya, Kenya, Nigeria, and Algeria. These countries receive higher numbers of air and coastal travelers and serve as transport hubs to other parts of Africa. In addition, South Africa, Morocco, Tunisia, Egypt, Ethiopia, and Libya are all located around the continent’s fringes and are hence border countries. Between January and April 2020 alone, over 2,000,000 international travels were recorded in South Africa and Ethiopia, while Nigeria and Kenya had recorded over 1,000,000 international travelers. Together, these four countries accounted for 47.9% of travel originating from countries where infections had been confirmed [40]. Similarly, the comparably low cases recorded in Mauritius, Seychelles, Comoros, and Sao Tome and Principe may not be unconnected to the fact that they are island countries separated from others by water.

The number of recoveries in each region has remained significantly higher than the number of deaths recorded since the onset of the pandemic in Africa, with the highest recovery rate observed in the Western African region between 2020 and 21 June 2021, ahead of Southern Africa, Central Africa, Northern Africa, and Eastern Africa. Elevated vitamin D levels have been associated with reduced severity and, hence, increased recovery from COVID-19 [41]. Adequate vitamin D level reduces the severity of thrombosis, pneumonia, and inflammation in COVID-19 patients, leading to shorter hospital stays [42]. Sunlight as a source of vitamin D is abundant in many African countries and may be a contributary factor to the high recovery rate. In addition, the continent of Africa has been hit by various other deadly infectious diseases in the past, such as Lassa fever, Ebola disease, and HIV. Hence, it may have been easier for such countries to apply lessons from previous public health responses to tackle the ongoing pandemic. For instance, a country such as Nigeria has had a robust system in place for the wild poliovirus, while the likes of DRC, Guinea, Liberia, and Sierra Leone have battle the Ebola virus disease before COVID-19. Studies have also reported that infection from SARS-CoV resulted in the formation of antibodies against SARS-CoV-2 [43] and other human coronaviruses [44]. Hence, although further research is required on this subject, it may be tenable to believe that antibody cross-reactivity from previous exposures to human coronaviruses on the continent may play a role in the observed milder cases and high recovery in Africa.

Despite the high recoveries, the CFR in Northern and Southern Africa exceeds the global CFR of 2.17%. This could be a pointer to a rising fatality on the continent since the advent of the second wave. The Africa CDC predicted a rise in fatality earlier in June 2021 as a trend was already observed in 21 out of 55 AU member countries where CFR had exceeded the global CFR [45]. CFR can aid in evaluating the quality of health systems and disease severity and identification of populations at risk of the disease [46]. As of 21 June 2021, the global CFR was 2.17%. This value falls below the expected range reported previously by Ghayda et al. (2020) [47]. Previous studies conducted in 2020 reported global CFR of 3.4% [48] and 3.1% [49]. CFR values for COVID-19 can be dynamic, with suggestions that it might be difficult to accurately determine [50]. The continued decline in CFR observed from June 2020 could be explained by the increased testing rate around that period compared to the earlier days of the pandemic when testing kits were in shorter supply. With more testing and a better understanding of the pandemic, fewer fatalities have resulted even though cumulative cases increased daily. Detection of asymptomatic individuals allows for early management of the disease and reduces the chances of it degenerating into a more severe stage that could lead to death. Globally, fewer than one-third of critically ill patients who required intensive care ended in fatality, whereas an average was nearly half of these patients ended in fatality in some African countries. This higher fatality among critically ill patients may not be unrelated to the absence of efficient, adequate healthcare in many countries of the continent [51]. During the earlier days of the pandemic in Africa, for instance, Kenya had only about 200 units of intensive care beds to serve its over 50 million population compared to 34 beds per 100,000 persons in the United States of America [52]. This is further compounded by poor infrastructures, such as the absence of good roads for interconnection between rural and urban settlements where most healthcare is concentrated in most African countries. It is also not uncommon for patients to present for treatment at a health facility only when their case has become very critical, sometimes due to lack of resources to foot medical bills, or dependence on traditional remedies. This delay in seeking treatment coupled with the shortage of required critical care on the continent could have negatively influenced fatality rate especially among the critically ill COVID-19 patients in Africa. Data on mortality rates in Africa may also be influenced by the poor or in some cases, complete absence of vital birth and death records, as well as accurate records on cause of death. For instance, a recent survey reported only 10% of deaths were registered in Africa, compared to 98% in Europe [53]. 

The WHO had previously warned that Africa could become the new epicenter of the pandemic with a projected 300,000 deaths and 30,000,000 persons likely to become poor due to the COVID-19 pandemic [54], possibly due to the weakening economy, poor state of healthcare, and existing diseases that increase the odds of mortality. This has, however, in general, not entirely been the case. Although the disease spreads easily, the mortality is lower compared to previous respiratory viral infections such as SARS-CoV (10%) and MERS-CoV (37%) [55]. Among the WHO regions, mortality per 100,000 persons was higher in America and Europe than in other regions, especially in the Western Pacific and African regions. In addition, mortality per 100,000 persons in relation to the population of the 20 most-affected countries in Africa still appears lower than most parts of the world, the highest being in Tunisia and South Africa as of the time of this study. Initially, the low incidence of the disease on the continent of Africa was being assumptively linked with the hot temperature of most countries within the continent and to possible genetic resistance by Africans [8]. This position is supported by the modelling study of Wang et al. (2021) [56] in the USA and China, which reported that COVID-19 transmission is suppressed by high temperature and relative humidity. A systematic review that studied how ambient environmental conditions influence mortality in COVID-19 also supported this assertion [57]. It is now agreed that the overall young age of Africa’s population has played a significant role in the low mortality observed. An earlier report has shown that the risk of mortality from COVID-19 increases by 11% per year of age compared to 10% for all-cause mortality [58]. Furthermore, 70% of COVID-19 related deaths in the United States of America occurs in people aged 70 years and above. It is estimated that 62% of the Africa ‘s population are 25 years or younger, with the median age on the continent being 19.4 years. Only 3% of Africans are 65 years or older compared to 18% in Europe and America [59,60]. A study of COVID-19 age-mortality in developing countries by Demombynes (2020) [61] revealed a higher death rate due to COVID-19 in high-income countries (HIC) than in low- and middle-income countries (LMIC). According to data obtained from 26 different countries, only 37% of deaths occurred in patients aged at least 70 years in LMIC, compared to 87% in HIC. In the HICs, mortality was 12.6 times higher in patients above 70 years than those between 50 and 59 years as compared to just 3.5 times in LMICs. Although the differences in COVID-19 cases and fatality between a continent such as Africa and other economically advantaged continents may not be explained by a single factor, it is obvious that age as a population demographic factor may be playing a major role.

## 5. Conclusions

Epidemiological trends in cases, testing rates, and fatalities per population varied based on regions and may be influenced by several factors, including the age of the general population, testing capacities of individual countries, and genetic makeup. The rate of infection and fatality in Africa is still quite low compared to other regions of the world. Still, the true state of the pandemic in Africa may have been masked by low testing rates and the lack of adequate surveillance systems for tracking community spread. Therefore, increased testing rates are required on the continent. There is a likelihood of increased incidence in Africa, going by the increasing CFR within the continent and the emergence of the new Delta variant first reported in India now being in some African countries. The world is truly a global village and is now more connected than it has ever been. The implication is that what begins elsewhere can travel around the world faster than ever imagined, including Africa. COVID-19 may not be the last such unfriendly visit from a highly infectious disease agent. Hence, Africa needs to deliberately build up a more robust and efficient healthcare system while also investing more into scientific research and development. Stringent measures are required to halt disease spread and prevent high fatality on the continent. Vaccination also needs to be ramped up, as has been done in many other countries of the world.

## Figures and Tables

**Table 1:** Top 20 countries in Africa with highest COVID-19 infections *.

Country	Total Cases	Total Deaths	Total Tests	CFR	C/100K	D/100K	Test/M	Population
South Africa	1,832,479	58,795	12,586,342	3.21	3090.18	99.15	212,248.60	59,308,690
Morocco	526,737	9244	6,192,757	1.75	1427.47	25.05	167,825.39	36,910,560
Tunisia	385,428	14,118	1,619,741	3.66	3266.34	119.64	137,266.19	11,818,619
Egypt	277,797	15,898	2,977,183	5.72	271.55	15.54	29,102.47	102,334,404
Ethiopia	275,318	4286	2,828,170	1.56	239.41	3.73	24,592.78	114,963,588
Libya	191,038	3178	1,081,611	5.66	2768.67	46.06	156,686.67	6,871,292
Kenya	179,293	3461	1,907,096	1.93	333.26	6.43	35,447.88	53,771,296
Nigeria	167,292	2118	2,266,591	1.27	81.17	1.03	10,997.53	206,139,589
Algeria	136,294	3772	230,553	2.77	310.46	8.59	5251.78	43,854,044
Zambia	130,631	1691	1,777,727	1.29	709.95	9.19	96,615.60	18,383,955
Ghana	95,059	794	1,241,303	0.84	305.66	2.55	39,913.28	31,072,940
Cameroon	80,487	1320	1,751,774	1.64	303.72	4.98	66,104.68	26,545,863
Namibia	75,766	1179	496,906	1.56	3030.64	47.16	198,762.40	2,540,905
Uganda	72,679	680	1,266,601	0.94	159.04	1.49	27,715.56	45,741,007
Mozambique	72,577	848	577,991	1.17	231.88	2.71	18,466.17	32,866,272
Botswana	64,021	926	1,230,113	1.45	2667.54	38.58	512,547.08	2,354,627
Cote D’Ivoire	48,047	308	690,236	0.64	182.00	1.17	26,145.30	26,378,274
Senegal	42,437	1158	562,497	2.73	254.11	6.93	33,682.46	16,743,927
Zimbabwe	42,195	1685	617,919	3.99	283.19	11.31	41,471.07	14,862,924
Madagascar	41,998	888	209,633	2.11	151.62	3.21	7567.98	27,691,018

DRC, Democratic Republic of Congo; CFR, case fatality ratio; 100k, 100 000; Test/M, tests per million population. * Based on AU CDC data of 21 June 2021. Source: https://africacdc.org/covid-19/ (accessed on 22 June 2021).

## Data Availability

Publicly available datasets were analyzed in this study. This data can be found here: https://www.who.int/emergencies/diseases/novel-coronavirus-2019/situation-reports (accessed on 22 June 2021; https://covid19.who.int/ (accessed on 18 August 2021); and https://africacdc.org/covid-19/ (accessed on 22 June 2021).
